# Association between triglyceride-glucose index and major adverse cardiovascular events in patients with intermediate coronary stenosis: a prospective cohort study

**DOI:** 10.3389/fendo.2025.1663832

**Published:** 2025-11-06

**Authors:** Hui-juan Li, Lei-guang Zhang, Haobo Xu, Yao-xin Wang, Shuai Liu, Jie-yun Liu

**Affiliations:** 1 Department of Cardiology, Kaifeng Central Hospital, Henan Medical University Affiliated Kaifeng Central Hospital, Kaifeng, Henan, China; 2 Kaifeng Key Laboratory of Clinical Medicine, Kaifeng, Henan, China; 3 Kaifeng Engineering Technology Research Center for Intelligent Diagnosis and Treatment of Cardiovascular Diseases, Kaifeng, Henan, China; 4 Department of Anesthesiology, Kaifeng 155 Hospital of China RongTong Medical Healthcare Group Co.Ltd., Kaifeng, Henan, China

**Keywords:** intermediate coronary stenosis, MACE, prognosis, TyG index, risk factors

## Abstract

**Objective:**

This study aimed to evaluate the correlation between the triglyceride-glucose (TyG) index and the incidence of major adverse cardiovascular events (MACE) in patients with intermediate coronary stenosis.

**Methods and results:**

A prospective cohort study was conducted involving 217 patients diagnosed with intermediate coronary stenosis confirmed by coronary angiography at Kaifeng Central Hospital. Patients were stratified into quartiles based on the TyG index and were followed for a median period of 858 days. During the follow-up, 35 patients experienced MACE. Kaplan-Meier survival analysis, adjusted for confounding variables in Model 3, demonstrated a significantly elevated risk of MACE in the highest TyG quartile compared with those in the lowest quartile (log-rank *p* = 0.015). The hazard ratio (HR) for MACE in the highest quartile was 1.87 (95% CI: 1.23–2.18; *p* = 0.005). Restricted cubic spline analysis demonstrated that the TyG index became a significant risk factor for MACE when exceeding a value of 10.19, with an overall positive trend in risk as the TyG index increased (nonlinear test *p* = 0.221). Exploratory subgroup analyses indicated that, when assessed as a continuous variable, the TyG index was significantly associated with a higher incidence of MACE among male patients (HR = 1.57; 95% CI: 1.05–2.36; *p* = 0.047), those aged over 65 years (HR = 1.25; 95% CI: 1.18–3.10; *p* = 0.027), and those with diabetes mellitus (HR = 1.91; 95% CI: 1.18–3.10; *p* = 0.021).

**Conclusion:**

The TyG index was independently correlated with an increased incidence of MACE in patients with intermediate coronary stenosis. Subgroup analyses indicated that this correlation was particularly pronounced in patients with diabetes mellitus.

## 1 Background

Coronary angiography (CAG), although widely used in clinical practice, presents notable limitations in the assessment of intermediate coronary artery stenosis. Accurate risk stratification and management of such lesions are essential to achieving an optimal balance between the risks associated with over-treatment and those of undertreatment. Lesions characterized by intermediate stenosis that do not undergo revascularization may still confer a substantial long-term cardiovascular risk, thereby necessitating careful surveillance (1). Enhancing risk stratification in patients with intermediate coronary artery stenosis remains a significant clinical challenge. Early identification of modifiable risk factors is essential for optimizing the management of coronary atherosclerosis (2, 3). Insulin resistance (IR) has been recognized as a key contributor to the pathogenesis of cardiovascular diseases (4, 5). Previous studies have demonstrated an association between the triglyceride-G (TyG) index and cardiovascular risk in patients with coronary artery disease (CAD) (5). However, the prognostic significance in patients with intermediate coronary stenosis has not been clearly established (6). Although prior studies have demonstrated that the association between the TyG index and cardiovascular outcomes may vary across patient subgroups, comprehensive analyses accounting for variables such as age, sex, and diabetes mellitus (DM) status remain limited (7). This prospective observational study was conducted to investigate the correlation between the TyG index and clinical outcomes in patients with angiographically confirmed intermediate coronary stenosis.

## 2 Participants and methods

### 2.1 Data collection

Baseline demographic data, clinical history, laboratory parameters, echocardiographic findings, and medication information were extracted from the electronic medical records at Kaifeng Central Hospital. Comorbidities including hypertension, DM, heart failure (HF), cerebrovascular disease, atrial fibrillation, and valvular heart disease were identified based on documented history and supporting diagnostic findings.

Laboratory indicators included fasting blood glucose (FBG), glycated hemoglobin (HbA1c), total cholesterol (TC), triglycerides (TG), low-density lipoprotein cholesterol (LDL-C), homocysteine (HCY), uric acid (UA), estimated glomerular filtration rate (eGFR), serum creatinine (Scr), alanine aminotransferase (ALT), and N-terminal pro-B-type natriuretic peptide (NT-proBNP). All parameters were measured using fasting venous blood samples collected after an overnight fast of at least 8 hours during the first hospital admission. Body mass index (BMI) was calculated as weight in kilograms divided by height in meters squared (kg/m²). The TyG index was calculated using the formula: TyG = ln [TG (mg/dL) × FBG (mg/dL)/2].

### 2.2 Study design

This single-center, prospective cohort study was conducted in the Department of Cardiology at Kaifeng Central Hospital. A total of 415 patients admitted between June 2020 and March 2023 with a new diagnosis of coronary atherosclerotic heart disease who underwent CAG were screened for eligibility. Intermediate coronary stenosis was defined as angiographic luminal narrowing between 50% and 70%. Among the screened cohort, 153 patients were excluded due to missing fasting TG and/or FBG values or suboptimal angiographic imaging attributable to factors such as calcification, vessel tortuosity, or poor image quality. An additional 45 patients were lost to follow-up. Finally, 217 patients were eligible for inclusion in the final analysis.

Exclusion Criteria: Patients were excluded if any of the following criteria were met: 1) Presence of a most severe stenotic lesion measuring < 40% or > 70% on CAG; 2) History of myocardial infarction; 3) Prior coronary revascularization, including percutaneous coronary intervention (PCI), percutaneous transluminal coronary angioplasty, or coronary artery bypass grafting; 4) Cardiogenic shock or HF classified as New York Heart Association Class III or IV HF; 5) Diagnosis of primary cardiomyopathy; 6) Presence of systemic illnesses including hypertensive emergencies, diabetic ketoacidosis, hyperthyroidism, acute exacerbation of chronic obstructive pulmonary disease, renal failure, severe coagulopathy, or active gastrointestinal bleeding; 7) Estimated life expectancy of less than 1 year; 8) Non-adherence to prescribed medications.

**Figure 1:**
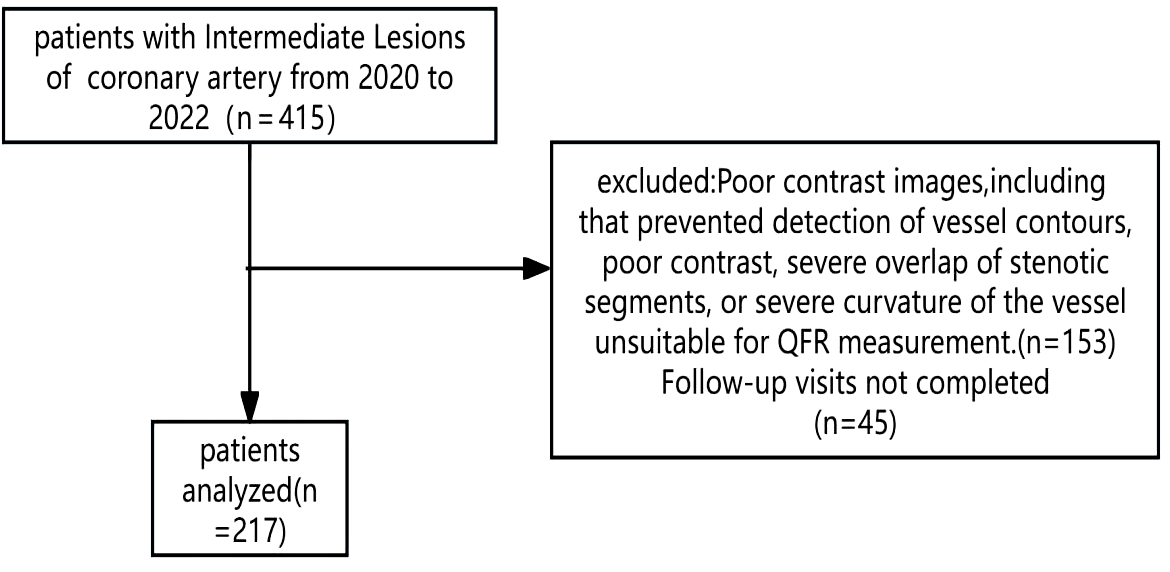
The flowchart of patients’ selection.

A total of 271 patients were included in this study (**Figure 1**). This study was approved by the Ethics Committee of Kaifeng Central Hospital (2021KS-LW009). Participants were followed until November 30, 2024.

### 2.3 Therapeutic interventions

In this study, statins were administered at the recommended standard doses according to the guidelines: atorvastatin 20mg/day or rosuvastatin 10mg/day. Under the condition of no contraindications, continuous treatment was maintained. LDL target achievement was defined as reducing to below 1.4 mmol/L or achieving a reduction of more than 50% compared to the baseline level. This study focused on the drug treatment for patients with borderline coronary artery lesions. SGLT-2 inhibitors were not routinely used. For patients with indications for SGLT-2 inhibitors, dapagliflozin was preferred, with an initial dose of 5mg/day. If there were no adverse reactions, the dose was increased to the standard dose of 10mg/day for maintenance.

### 2.4 Statistical analysis

Participants were stratified into quartiles based on the TyG index: Q1: TyG < 9.84, Q2: 9.84 ≤ TyG < 10.19, Q3: 10.19 ≤ TyG < 10.82, and Q4: TyG > 10.82. Comparative analyses of baseline characteristics and follow-up outcomes were conducted across TyG index quartiles (**Table 1**). Categorical variables are expressed as frequencies and percentages and compared using Pearson’s chi-squared test or Fisher’s exact test. Continuous variables are expressed as mean ± standard deviation or interquartile range, depending on distribution, and compared using analysis of variance or Kruskal-Wallis test, as appropriate.

**Table 1:** Comparison of baseline characteristics across TyG index quartiles.

Variables	Total (n = 217)	Q1 (n = 53)	Q2 (n = 55)	Q3 (n = 54)	Q4 (n = 55)	T or F value	*P-*value
Sex, n (%)						2.526	0.471
Male	117 (53.9)	25 (47.2)	33 (60)	27 (50)	32 (58.2)		
Female	100 (46.1)	28 (52.8)	22 (40)	27 (50)	23 (41.8)		
Age Mean ± SD	64.1 ± 9.8	65.5 ± 10.2	64.1 ± 10.4	64.3 ± 7.3	62.8 ± 10.8	0.693	0.557
BMIKg/m2., Mean ± SD	24.8 ± 3.2	23.8 ± 3.5	24.7 ± 2.8	25.1 ± 3.4	25.5 ± 3.1	2.773	0.042
Smoking, n (%)						2.404	0.493
No	146 (67.3)	40 (75.5)	37 (67.3)	34 (63)	35 (63.6)		
Yes	71 (32.7)	13 (24.5)	18 (32.7)	20 (37)	20 (36.4)		
Drinking, n (%)						3.746	0.29
No	145 (66.8)	41 (77.4)	35 (63.6)	33 (61.1)	36 (65.5)		
Yes	72 (33.2)	12 (22.6)	20 (36.4)	21 (38.9)	19 (34.5)		
Hypertension, n (%)						1.552	0.67
No	61 (28.1)	18 (34)	14 (25.5)	13 (24.1)	16 (29.1)		
Yes	156 (71.9)	35 (66)	41 (74.5)	41 (75.9)	39 (70.9)		
Diabetes mellitus, n (%)						4.334	0.228
No	168 (77.4)	44 (83)	46 (83.6)	39 (72.2)	39 (70.9)		
Yes	49 (22.6)	9 (17)	9 (16.4)	15 (27.8)	16 (29.1)		
Comorbid heart failure, n (%)						Fisher	0.88
No	199 (91.7)	47 (88.7)	51 (92.7)	50 (92.6)	51 (92.7)		
Yes	18 ( 8.3)	6 (11.3)	4 (7.3)	4 (7.4)	4 (7.3)		
Atrial fibrillation, n (%)						1.46	0.692
No	190 (87.6)	46 (86.8)	46 (83.6)	48 (88.9)	50 (90.9)		
Yes	27 (12.4)	7 (13.2)	9 (16.4)	6 (11.1)	5 (9.1)		
Valvular heart disease, n (%)						Fisher	0.092
No	202 (93.1)	46 (86.8)	50 (90.9)	53 (98.1)	53 (96.4)		
Yes	15 ( 6.9)	7 (13.2)	5 (9.1)	1 (1.9)	2 (3.6)		
Cerebrovascular disease, n (%)						3.052	0.384
No	180 (82.9)	44 (83)	42 (76.4)	48 (88.9)	46 (83.6)		
Yes	37 (17.1)	9 (17)	13 (23.6)	6 (11.1)	9 (16.4)		
Chronic renal insufficiency, n (%)						Fisher	0.62
No	215 (99.1)	52 (98.1)	54 (98.2)	54 (100)	55 (100)		
Yes	2 ( 0.9)	1 (1.9)	1 (1.8)	0 (0)	0 (0)		
LDL.C.mmol/L, Mean ± SD	2.6 ± 0.8	2.6 ± 0.8	2.7 ± 0.8	2.4 ± 0.8	2.6 ± 0.8	0.768	0.513
TC.mmol/L, Mean ± SD	4.1 ± 1.0	4.2 ± 0.9	4.1 ± 1.0	3.9 ± 0.9	4.1 ± 1.0	0.646	0.586
FBG.mmol/L, Median (IQR)	5.3 (4.8, 6.1)	4.8 (4.4, 5.5)	5.3 (4.9, 5.9)	5.3 (4.9, 6.3)	5.7 (5.2, 6.7)	17.107	< 0.001
TG.mmol/L, Median (IQR)	1.2 (0.9, 1.8)	0.8 (0.7, 1.0)	1.1 (1.0, 1.2)	1.5 (1.3, 1.8)	2.0 (1.2, 2.8)	60.64	< 0.001
NT.proBNP.pg/ml, Median (IQR)	89.3 (56.4, 233.9)	93.3 (56.7, 273.6)	83.2 (55.1, 215.5)	88.6 (56.1, 260.4)	93.2 (56.9, 213.4)	0.162	0.983
HbA1c.%, Median (IQR)	5.7 (5.3, 6.1)	5.7 (5.3, 6.1)	5.7 (5.3, 6.0)	5.7 (5.2, 6.4)	5.6 (5.3, 6.0)	0.494	0.92
ALT.U/L, Median (IQR)	16.0 (12.0, 20.0)	17.0 (12.0, 22.0)	14.0 (11.0, 19.5)	15.5 (11.0, 21.8)	16.0 (13.0, 20.0)	2.546	0.467
Hcy, Median (IQR)	14.3 (12.5, 17.5)	14.3 (12.5, 17.7)	15.0 (13.0, 21.0)	14.2 (12.1, 17.4)	14.0 (12.7, 15.8)	3.283	0.35
eGFR, Median (IQR)	96.0 (87.0, 111.0)	96.0 (86.0, 110.0)	96.0 (88.0, 105.5)	95.0 (80.2, 114.8)	99.0 (90.5, 118.0)	4.078	0.253
Scr.umol/L, Median (IQR)	63.0 (56.0, 75.0)	63.0 (55.0, 77.0)	64.0 (57.5, 73.0)	66.5 (59.0, 80.8)	60.0 (56.0, 68.0)	6.254	0.1
UA.mg/dl, Median (IQR)	295.0 (242.0, 338.0)	298.0 (254.0, 373.0)	303.0 (252.5, 350.5)	291.0 (240.8, 332.5)	292.0 (228.5, 334.5)	1.753	0.625
LA.mm, Median (IQR)	37.0 (35.0, 40.0)	37.0 (35.0, 40.0)	37.0 (35.0, 42.0)	37.5 (35.0, 40.8)	37.0 (34.5, 40.0)	1.403	0.705
LVEDD.mm, Median (IQR)	45.0 (42.0, 49.0)	45.0 (43.0, 49.0)	45.0 (41.5, 48.0)	46.5 (43.0, 49.0)	45.0 (42.0, 48.5)	3.474	0.324
EF.%, Median (IQR)	69.0 (64.0, 73.0)	70.0 (63.0, 73.0)	68.0 (65.0, 73.5)	69.0 (65.0, 72.0)	68.0 (62.0, 72.0)	0.739	0.864
Whether it is single-vessel disease, n (%)						0.15	5.313
No	72 (33.2)	23 (43.4)	20 (36.4)	16 (29.6)	13 (23.6)		
Yes	145 (66.8)	30 (56.6)	35 (63.6)	38 (70.4)	42 (76.4)		
QFR.cut, n (%)						0.662	1.587
QFR≥0.8	184 (84.8)	47 (88.7)	45 (81.8)	47 (87)	45 (81.8)		
QFR<0.8	33 (15.2)	6 (11.3)	10 (18.2)	7 (13)	10 (18.2)		
MACE, n (%)						10.613	0.014
0	182 (83.9)	48 (90.6)	50 (90.9)	45 (83.3)	39 (70.9)		
1	35 (16.1)	5 (9.4)	5 (9.1)	9 (16.7)	16 (29.1)		

TC, total cholesterol; LDL-C, low-density lipoprotein cholesterol; BMI, body mass index; FBG, fasting blood glucose; HCY, homocysteine; UA, uric acid; GFR, glomerular filtration rate; Scr, creatinine; ALT, alanine aminotransferase; TG: triglycerides; HbA1c, glycosylated hemoglobin; NT-proBNP, precursor of N-terminal brain natriuretic peptide; LVEDD, left ventricular end-diastolic diameter; LAD, left anterior descending; LVEF, left ventricular ejection fraction; QFR, quantitative flow ratio; MACE, major adverse cardiovascular event.

**Figure 2:**
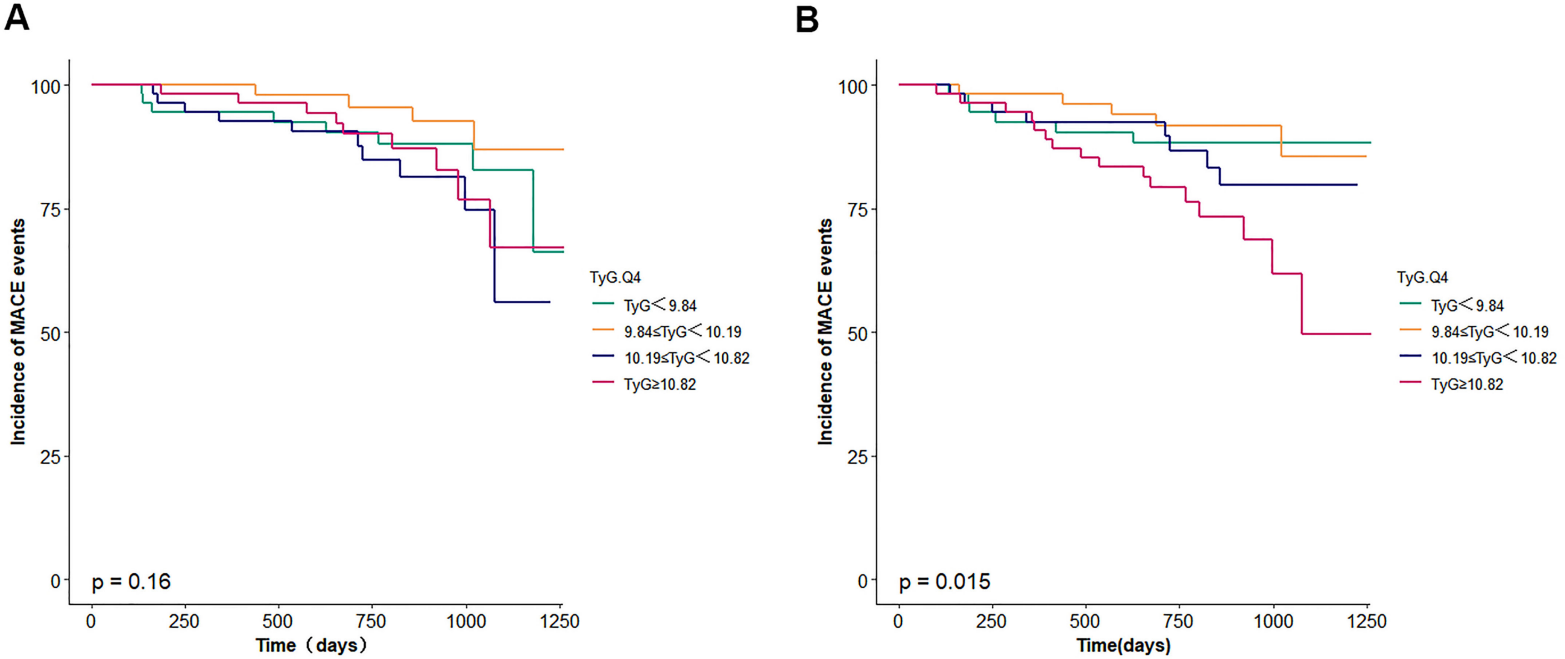
**(A)** Survival curves for incidence of MACE events. TyG, triglyceride glucose. **(B)** adjusted survival curves for incidence of MACE events. TyG, triglyceride glucose. The model was adjusted for age, body mass index, sex, smoking, diabetes mellitus, chronickidney disease, haemoglobin, urea, serum creati-nine, N-terminal pro-B type natriuretic peptide, total cholesterol, low-density lipoprotein cholesterol, triglyceride, left ventricular ejectionfraction.

**Figure 3:**
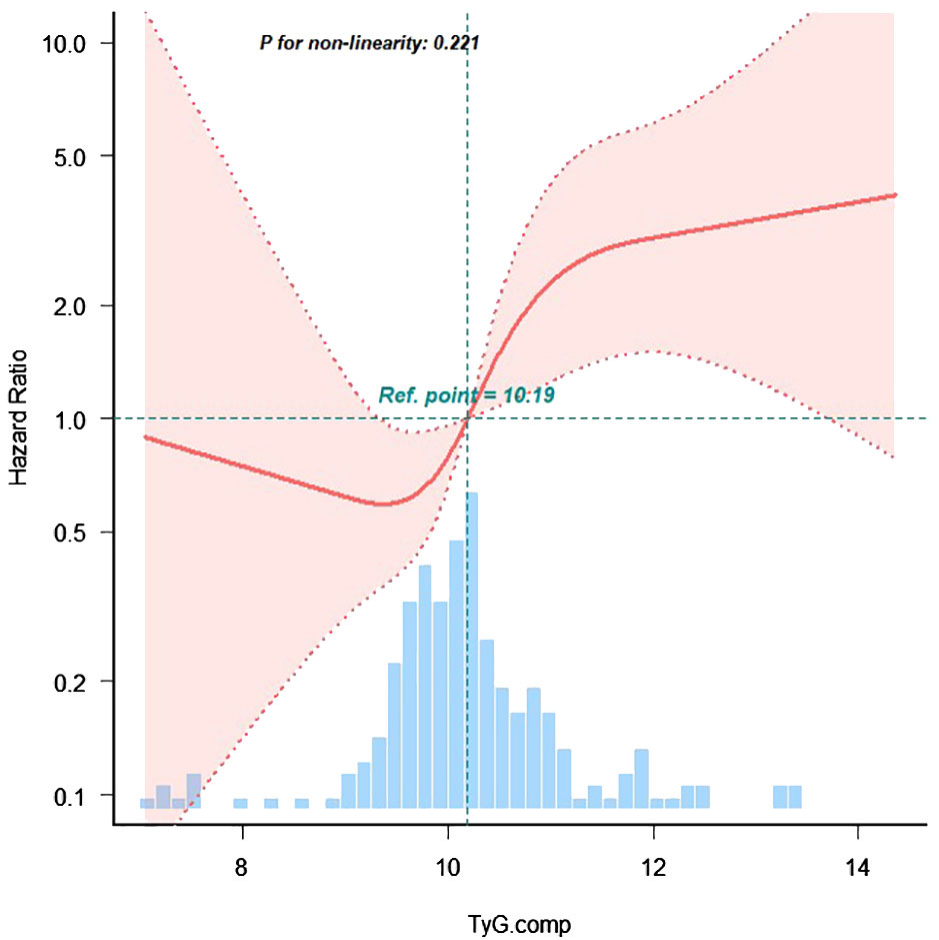
The restricted cubic spline of TyG index and incidence of MACE events. The blue columns present the distribution density of the TyG index. The reference point 10.19 repreents the mean value of TyG index. The redline shows the hazard ratio. The red shaded area represents the 95% con-fidence interval. The model was adjusted for age, body mass index, sex, smoking, diabetes mellitus, chronickidney disease, haemoglobin, urea, serum creati-nine, N-terminal pro-B type natriuretic peptide, total cholesterol, low-density lipoprotein cholesterol, triglyceride, left ventricular ejectionfraction.

Survival analysis was conducted to evaluate the correlation between TyG index quartiles and incidence of major adverse cardiovascular events (MACE). Unadjusted and multivariate-adjusted Kaplan-Meier survival curves were constructed (**Figures 2**, **3**). Multivariate Cox proportional hazards regression models were applied to assess the correlation between the TyG index and MACE. Covariates included in the models were selected based on clinical relevance and significance in univariate analyses. Hazard ratios (HRs) and 95% confidence intervals (CIs) were reported. The models (**Table 2**) were adjusted as follows:

**Table 2:** Cox proportional hazard(HR) for all-cause mortality.

Tyg	Events,n(%)	Model 1a		Model2b		Model3b	
		HR(95%CI)	*P*	HR(95% CI)	*P*	HR(95% CI)	*P*
Quartile1	424(14)	1(Ref)		1(Ref)		1(Ref)	
Quartile2	439(14.5)	1.04 (0.89~1.21)	0.209	1.1 (0.93~1.31)	0.349	1.03 (0.47~1.21)	0.615
Quartile3	584(19.3)	1.27 (0.91~1.7)	0.515	1.37 (0.61~1.6)	0.233	1.54 (1.09~1.84)	0.801
Quartile4	554(18.3)	1.89 (1.02~2.41)	0.219	2.14 (1.76~2.61)	0.308	1.87 (1.23~2.18)	0.005
Trend.test		1.3 (1.25~1.36)	0.297	1.31 (1.22~1.39)	0.425	1.45 (1.14~1.82)	0.001

- Model 1: Adjusted for age, sex, and BMI;
- Model 2: Model 1 plus adjustment for history of hypertension, DM, cerebrovascular disease, chronic kidney disease, atrial fibrillation, and valvular heart disease;
- Model 3: Model 2 plus adjustment for biochemical and echocardiographic variables, including FBG, HbA1c, HCY, UA, LDL-C, eGFR, Scr, ALT, TG, NT-proBNP, left ventricular end-diastolic diameter (LVEDD), left atrial diameter (LAD), and left ventricular ejection fraction (LVEF).

**Figure 4:**
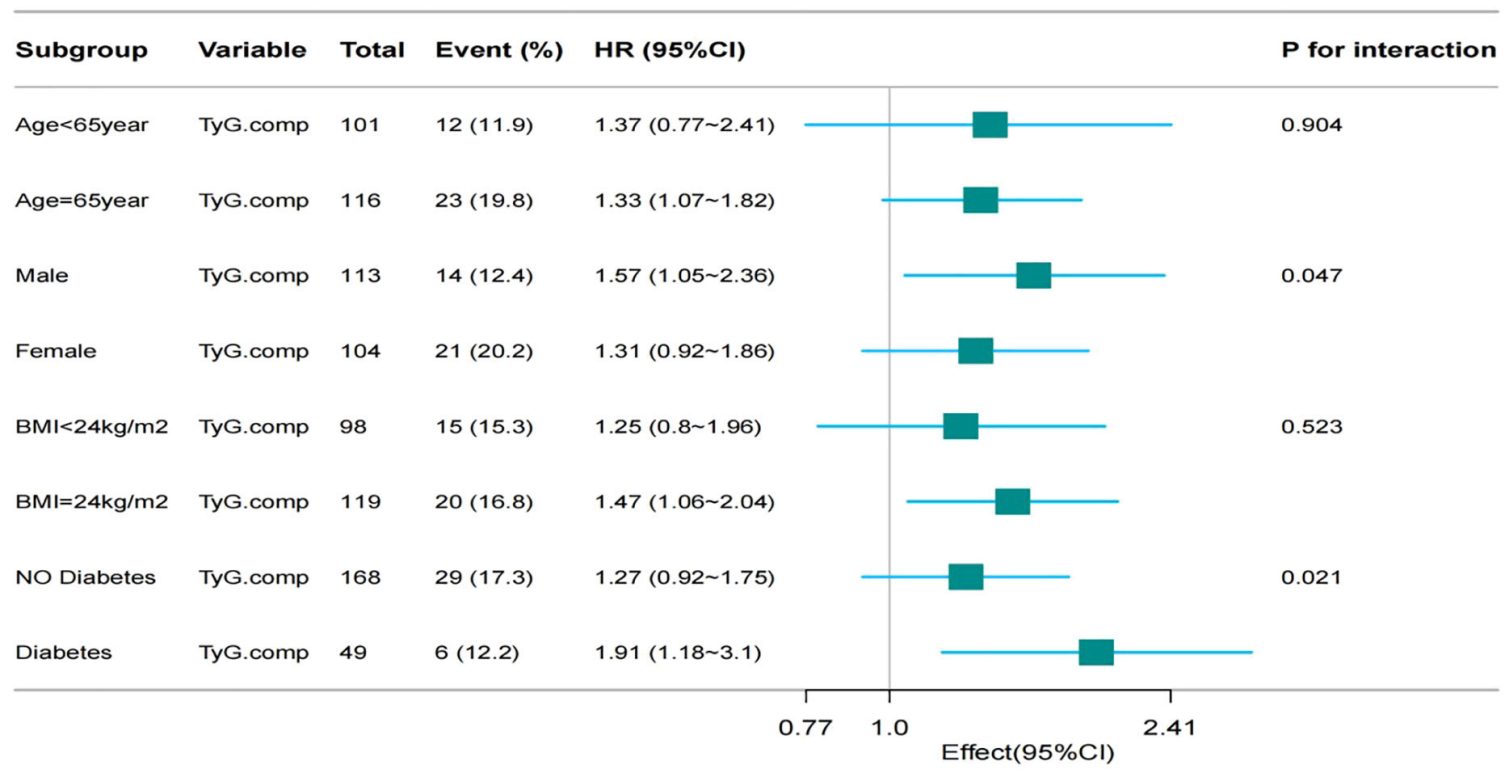
Subgroup analysis for association between TyG index and incidence of MACE events.

Restricted cubic spline analysis was performed to examine the non-linear relationship between TyG index and MACE incidence among patients with HF (**Figure 4**). Subgroup analyses were conducted based on age (< 65 vs. ≥ 65 years), sex (male vs. female), BMI (< 24 vs. ≥ 24 kg/m²), and DM status (presence vs. absence). Interaction terms were included to assess potential effect modification across subgroups.

All statistical analyses were performed using R software version 4.1.0, and SPSS software, version 27.0 for Windows. The multiple imputation using the chained equations (MICE) method was applied for the relationships between variables and provides robust estimates by generating multiple plausible values for missing data. A two-tailed *p* value < 0.05 was considered statistically significant.

## 3 Results

### 3.1 Baseline characteristics

A total of 217 patients with intermediate coronary artery stenosis were included in the analysis and stratified into four groups according to TyG index quartiles. The median duration of follow-up was 858 days, during which 35 patients (16.1%) experienced MACE. The mean age of the cohort was 64.1 ± 9.8 years, and 117 patients (53.9%) were male.

Comparative analysis across the four TyG index quartiles demonstrated no statistically significant differences in demographic or clinical characteristics, including sex, age, and history of hypertension, DM, chronic renal insufficiency, and dyslipidemia. Similarly, there were no significant differences in key biochemical or echocardiographic indices, including FBG, HbA1c, HCY, UA, LDL-C, eGFR, serum Scr, ALT, NT-proBNP, LVEDD, LAD, and LVEF (*p* > 0.05 for all).

### 3.2 Association between TyG index and MACE

During the follow-up period, a total of 35 MACE events occurred, with incidence rates as follows: 5 events (9.4%) in the first quartile (Q1), 5 events (9.1%) in Q2, 9 events (16.7%) in Q3, and 16 events (29.1%) in Q4. Although unadjusted Kaplan-Meier survival analysis did not demonstrate statistically significant differences in event-free survival across TyG quartiles, adjusted survival analysis accounting for confounding variables in Model 3 indicated a statistically significant association between higher TyG index quartiles and increased MACE risk (*p* = 0.015).

Multivariable Cox proportional hazards regression analysis showed that the highest TyG index group (Q4) was associated with a greater risk of MACE compared with those in the lowest quartile (**Table 2**), as follows: Model 1 (adjusted for age, sex, and BMI): HR = 1.89, 95% CI: 1.02–2.41, *p* = 0.219; Model 2 (further adjusted for comorbidities including hypertension, DM, HF, atrial fibrillation, chronic kidney disease, valvular heart disease): HR = 2.14, 95% CI: 1.76–2.61, *p* = 0.308. However, statistically significant differences between quartiles were not observed in Models 1 and 2 except for the highest TyG index group (Q4).

In Model 3, which included additional adjustment for laboratory and echocardiographic parameters (FBG, HbA1c, HCY, UA, LDL-C, GFR, Scr, ALT, TG, NT-proBNP, LVEDD, LAD, and LVEF), both Q3 and Q4 were significantly correlated with an increased risk for MACE Q3: HR = 1.54, 95% CI: 1.09–1.84, *p* = 0.801; Q4: HR = 1.87, 95% CI: 1.23–2.18, *p* = 0.005.

A trend analysis confirmed a positive correlation between the TyG index and MACE incidence (*p* for trend < 0.001). Restricted cubic spline regression further demonstrated that, when the TyG index was analyzed as a continuous variable, the risk of MACE increased progressively for TyG values exceeding 10.19 (*p* for non-linearity = 0.221), indicating a linear dose-response relationship.

### 3.3 Exploratory subgroup analyses

Subgroup analyses identified a statistically significant association between the TyG index and the incidence of MACE in several participant subgroups: Older adults (≥ 65 years): HR = 1.33, 95% CI: 1.07–1.82, *p* = 0.904l; male sex: HR = 1.57, 95% CI: 1.05–2.36, *p* = 0.047; patients with DM: HR = 1.91, 95% CI: 1.18–3.10, *p* = 0.021.

No statistically significant association was observed in subgroup analyses stratified by BMI. These findings indicate that the predictive value of the TyG index may be more pronounced in specific clinical subgroups, particularly in those with established metabolic dysfunction or male sex (**Figure 4**).

## 4 Discussion

Intermediate coronary artery stenosis has emerged as a critical focus in cardiovascular research due to its diagnostic complexity and variable prognostic implications. The TyG index, a composite metabolic biomarker derived from fasting triglyceride and fasting glucose levels, has gained attention as a surrogate marker for IR and a potential predictor of cardiovascular outcomes (8–12). Previous studies have demonstrated that an elevated TyG index may reflect the progression of subclinical CAD. For example, the TyG index has been shown to predict both the severity of coronary lesions and the occurrence of MACE (13). Among patients without conventional cardiovascular risk factors, the TyG index has been proposed as a marker for early subclinical atherosclerotic disease (14). Additional evidence indicates that elevated TyG index levels are independently associated with adverse cardiovascular and cerebrovascular outcomes following percutaneous PCI, particularly in female patients (15). In the context of intermediate coronary stenosis, the TyG index may hold significant prognostic value. A previous study demonstrated that an elevated TyG index serves as a predictor of MACE within one year in patients with both end-stage renal disease and CAD, suggesting its utility in risk stratification within specific subpopulations of patients with CAD (16). Furthermore, another study proposed that integrating the TyG index with coronary plaque characteristics enhances the accuracy of predicting adverse cardiovascular outcomes following PCI (8).

Large-scale, database-driven cohort studies have validated the TyG index and its derivatives as cost-effective and accessible markers for cardio-metabolic risk assessment, which is particularly valuable in healthcare settings with limited resources. When combined with anthropometric indicators of obesity such as BMI (e.g., TyG-BMI) predictive performance of the TyG index can be further enhanced (17–23).

Nevertheless, the application of the TyG index has been subject to scrutiny. Its predictive reliability may be compromised in the context of comorbid conditions such as DM and hyperlipidemia (24). Notably, while the TyG index remains an independent predictor of coronary artery stenosis severity among patients without DM, its discriminative performance appears to be reduced in the DM population.

Evidence from multiple studies has demonstrated a positive correlation between the TyG index and both the prevalence of symptomatic CAD and established metabolic and behavioral risk factors (25–32). These findings support the use of the TyG index as a surrogate marker of atherosclerotic disease burden.

IR is recognized as a key contributor to atherosclerotic cardiovascular disease. As a surrogate indicator of IR, the TyG index reflects the extent of metabolic dysfunction. IR may promote atherosclerotic processes through several mechanisms:

Enhanced Inflammatory Response: IR is associated with chronic systemic inflammation, characterized by up-regulation of pro-inflammatory cytokines (e.g., interleukin-6, tumor necrosis factor-alpha) and acute-phase reactants such as C-reactive protein, which may contribute to arterial wall inflammation (3–7).

Dysregulated Lipid Metabolism: IR is commonly associated with dyslipidemia, characterized by hypertriglyceridemia and reduced HDL-C levels, both of which are known to accelerate atherogenesis (8, 9).

Endothelial Dysfunction: IR may impair endothelium-dependent vasodilatation, thereby exacerbating CAD progression.

Regarding plaque instability, particularly in cases of intermediate coronary stenosis, plaque vulnerability is a critical determinant of MACE occurrence. Elevated TyG index values have been associated with adverse plaque characteristics, including lipid-rich cores and thin fibrous caps, which are prone to rupture and precipitate acute coronary events (8). When combined with intravascular imaging modalities such as optical coherence tomography, the TyG index may improve prognostic assessment following PCI.

Among patients both with and without DM, a higher TyG index correlates with an increased risk of MACE, highlighting its role not only as a reflection of glycemic control but also as a comprehensive marker of metabolic syndrome encompassing risk factors such as dyslipidemia, obesity, and hypertension (7–9, 12).

A nonlinear dose-response correlation between the TyG index and MACE risk has been reported, wherein the risk increases steeply with rising TyG index values up to a certain threshold, beyond which a plateau in risk may occur (33–36). These findings indicate the presence of a threshold effect in the association between TyG index elevation and cardiovascular risk.

In summary, the TyG index is implicated in the risk of MACE among patients with intermediate coronary stenosis through multiple interconnected mechanisms, including IR, inflammation, lipid metabolism abnormalities, endothelial dysfunction, and plaque instability. Accordingly, routine monitoring of the TyG index may assist in identifying high-risk patients and informing the development of personalized therapeutic strategies.

While the TyG index demonstrates promise in the context of CAD, its clinical applicability and limitations require further elucidation. Future research should emphasize personalized medicine approaches and the integration of the TyG index with other cardiovascular biomarkers. Notably, reliance on baseline TyG index values alone may be insufficient to predict MACE in intermediate coronary stenosis. Longitudinal studies assessing the progression of stenosis and dynamic changes in the TyG index during follow-up are warranted.

Several limitations of this study should be acknowledged. First, the inherent selection bias and potential attrition associated with single-center observational studies may affect the generalizability of findings. Second, biochemical parameters were derived from a single fasting blood sample obtained at admission, introducing the possibility of measurement variability. Third, due to the limited original database collection, lifestyle factors such as dietary intake and physical activity, which may influence TyG index levels, were not assessed. To assess the potential impact of these factors, we had to conduct the sensitivity analysis. Lastly, comparative analyses between the TyG index and other established functional indicators, such as quantitative flow ratio or fractional flow reserve in predicting the prognosis of intermediate coronary stenosis were not performed.

## 5 Conclusion

In conclusion, the findings of this study demonstrate a significant positive correlation between the TyG index and the incidence of MACE among patient with intermediate coronary stenosis. Subgroup analyses indicate that this association is particularly evident in patients with DM. Further multicenter prospective cohort studies are essential to substantiate these observations and to delineate the role of the TyG index in cardiovascular risk stratification within this population.The residual confounding factors not detected in this article and the lack of continuous measurement of TyG values may have an impact on the observed one-year prognosis of critical coronary artery disease. In future studies, we will use repeated measurement methods and strengthen the refinement of drug use tracking to obtain more accurate and rigorous results.

## Data Availability

The raw data supporting the conclusions of this article will be made available by the authors, without undue reservation.

## References

[1] Tobis J Azarbal B Slavin L . Assessment of intermediate severity coronary lesions in the catheterization laboratory. J Am Coll Cardiol. (2007) 49:839–48. doi: 10.1016/j.jacc.2006.10.055, PMID: 17320741

[2] Bae JH Corban MT Seo YH Kim T Lee G Kwon TG . Ten-year clinical outcomes of an intermediate coronary lesion; prognosis and predictors of major adverse cardiovascular events. Int J Cardiol. (2020) 299:26–30. doi: 10.1016/j.ijcard.2019.06.076, PMID: 31281049

[3] Stary HC Chandler AB Glagov S Guyton JR Insull W Rosenfeld ME . A definition of initial, fatty streak, and intermediate lesions of atherosclerosis. A report from the Committee on Vascular Lesions of the Council on Arteriosclerosis, American Heart Association. Circulation. (1994) 89:2462–78. doi: 10.1161/01.cir.89.5.2462, PMID: 8181179

[4] Unger G Benozzi SF Perruzza F Pennacchiotti GL . Triglycerides and glucose index: a useful indicator of insulin resistance. Endocrinol Nutr. (2014) 61:533–40. doi: 10.1016/j.endonu.2014.06.009, PMID: 25174769

[5] Mahdavi-Roshan M Mozafarihashjin M Shoaibinobarian N Ghorbani Z Salari A Savarrakhsh A . Evaluating the use of novel atherogenicity indices and insulin resistance surrogate markers in predicting the risk of coronary artery disease: a case–control investigation with comparison to traditional biomarkers. Lipids Health Dis. (2022) 21:126. doi: 10.1186/s12944-022-01732-9, PMID: 36435770 PMC9701407

[6] Kim YK Kwon SH Seo YH Kim KH Kwon TG Bae JH . Angiographic predictors for repeated revascularization in patients with intermediate coronary lesions. Biomedicines. (2024) 12:2825. doi: 10.3390/biomedicines12122825, PMID: 39767731 PMC11672955

[7] Alizargar J Bai CH Hsieh NC Wu SV . Use of the triglyceride-glucose index (TyG) in cardiovascular disease patients. Cardiovasc Diabetol. (2020) 19 :8. doi: 10.1186/s12933-019-0982-2, PMID: 31941513 PMC6963998

[8] Park K Ahn CW Lee SB Kang S Nam JS Lee BK . Elevated tyG index predicts progression of coronary artery calcification. Diabetes Care. (2019) 42 :1569–73. doi: 10.2337/dc18-1920, PMID: 31182490

[9] Park GM Cho YR Won KB Yang YJ Park S Ann SH . Triglyceride glucose index is a useful marker for predicting subclinical coronary artery disease in the absence of traditional risk factors. Lipids Health Dis. (2020) 19 :7. doi: 10.1186/s12944-020-1187-0, PMID: 31937313 PMC6961240

[10] Gao A Liu J Hu C Liu Y Zhu Y Han H . Association between the triglyceride glucose index and coronary collateralization in coronary artery disease patients with chronic total occlusion lesions. Lipids Health Dis. (2021) 20 :140. doi: 10.1186/s12944-021-01574-x, PMID: 34689767 PMC8543811

[11] Zhao X Wang Y Chen R Li J Zhou J Liu C . Triglyceride glucose index combined with plaque characteristics as a novel biomarker for cardiovascular outcomes after percutaneous coronary intervention in ST-elevated myocardial infarction patients: an intravascular optical coherence tomography study. Cardiovasc Diabetol. (2021) 20 :131. doi: 10.1186/s12933-021-01321-7, PMID: 34183007 PMC8240222

[12] Darroudi S Mahdavizadeh V Mirzaei AH Esparham A Ahmadyar S Esmaily H . Triglyceride glucose index and triglyceride HDL ratio as predictors of coronary artery stenosis in diabetic and non-diabetic patients. Nutr Metab CARDIOVAS. (2024) 34 :1692–5. doi: 10.1016/j.numecd.2023.12.001, PMID: 38772847

[13] Siverio-Morales O Mora-Fernández C Hernández-Carballo C Martín-Núñez E González-Luis A Martín-Olivera A . Predictive value of triglyceride-glucose index for the evaluation of coronary artery disease severity and occurrence of major adverse cardiovascular events. Am J PHYSIOL-HEART C. (2025) 328:H14–20. doi: 10.1152/ajpheart.00684.2024, PMID: 39560964

[14] Yuan W Shao Y Zhao D Zhang B . Correlation analysis of lipid accumulation index, triglyceride-glucose index and H-type hypertension and coronary artery disease. PeerJ. (2023) 11:e16069. doi: 10.7717/peerj.16069, PMID: 37727694 PMC10506588

[15] Zou S Xu Y . Association of the triglyceride-glucose index and major adverse cardiac and cerebrovascular events in female patients undergoing percutaneous coronary intervention with drug-eluting stents: A retrospective study. Diabetes Res Clin PR. (2021) 181:109073. doi: 10.1016/j.diabres.2021.109073, PMID: 34592393

[16] Xie E Ye Z Wu Y Zhao X Li Y Shen N . The triglyceride-glucose index predicts 1-year major adverse cardiovascular events in end-stage renal disease patients with coronary artery disease. Cardiovasc Diabetol. (2023) 22:292. doi: 10.1186/s12933-023-02028-7, PMID: 37891651 PMC10612201

[17] Zhang Q Xiao S Jiao X Shen Y . The triglyceride-glucose index is a predictor for cardiovascular and all-cause mortality in CVD patients with diabetes or pre-diabetes: evidence from NHANES 2001-2018. Cardiovasc Diabetol. (2023) 22:279. doi: 10.1186/s12933-023-02030-z, PMID: 37848879 PMC10583314

[18] Liu C Liang D Xiao K Xie L . Association between the triglyceride-glucose index and all-cause and CVD mortality in the young population with diabetes. Cardiovasc Diabetol. (2024) 23:171. doi: 10.1186/s12933-024-02269-0, PMID: 38755682 PMC11097545

[19] Xiao S Zhang Q Yang HY Tong JY Yang RQ . The association between triglyceride glucose-body mass index and all-cause and cardiovascular mortality in diabetes patients: a retrospective study from NHANES database. Sci Rep. (2024) 14:13884. doi: 10.1038/s41598-024-63886-z, PMID: 38880806 PMC11180665

[20] Liu Q Zhang Y Chen S Xiang H Ouyang J Liu H . Association of the triglyceride-glucose index with all-cause and cardiovascular mortality in patients with cardiometabolic syndrome: a national cohort study. Cardiovasc Diabetol. (2024) 23:80. doi: 10.1186/s12933-024-02152-y, PMID: 38402393 PMC10893675

[21] Zhang Y Wang F Tang J Shen L He J Chen Y . Association of triglyceride glucose-related parameters with all-cause mortality and cardiovascular disease in NAFLD patients: NHANES 1999-2018. Cardiovasc Diabetol. (2024) 23:262. doi: 10.1186/s12933-024-02354-4, PMID: 39026233 PMC11264797

[22] Ding L Fu B Zhang H Dai C Zhang A Yu F . The impact of triglyceride glucose-body mass index on all-cause and cardiovascular mortality in elderly patients with diabetes mellitus: evidence from NHANES 2007-2016. BMC Geriatr. (2024) 24:356. doi: 10.1186/s12877-024-04992-5, PMID: 38649828 PMC11034154

[23] Dang K Wang X Hu J Zhang Y Cheng L Qi X . The association between triglyceride-glucose index and its combination with obesity indicators and cardiovascular disease: NHANES 2003-2018. Cardiovasc Diabetol. (2024) 23:8. doi: 10.1186/s12933-023-02115-9, PMID: 38184598 PMC10771672

[24] Hu B Wang Y Wang Y Feng J Fan Y Hou L . Association between Triglyceride-Glucose Index and risk of all-cause and cardiovascular mortality in adults with prior cardiovascular disease: a cohort study using data from the US National Health and Nutrition Examination Survey, 2007-2018. BMJ Open. (2024) 14:e084549. doi: 10.1136/bmjopen-2024-084549, PMID: 38969366 PMC11227790

[25] Yang M Shangguan Q Xie G Sheng G Yang J . Oxidative stress mediates the association between triglyceride-glucose index and risk of cardiovascular and all-cause mortality in metabolic syndrome: evidence from a prospective cohort study. Front Endocrinol (Lausanne). (2024) 15:1452896. doi: 10.3389/fendo.2024.1452896, PMID: 39229375 PMC11368748

[26] Zheng D Cai J Xu S Jiang S Li C Wang B . The association of triglyceride-glucose index and combined obesity indicators with chest pain and risk of cardiovascular disease in American population with pre-diabetes or diabetes. Front Endocrinol (Lausanne). (2024) 15:1471535. doi: 10.3389/fendo.2024.1471535, PMID: 39309107 PMC11412814

[27] Chen T Wan H Luo Y Chen L . Association of triglyceride-glucose-body mass index with all-cause and cardiovascular mortality among individuals with chronic kidney disease. Sci Rep. (2024) 14:20593. doi: 10.1038/s41598-024-71579-w, PMID: 39232126 PMC11375041

[28] Li S An L Fu Z Zhang W Liu H . Association between triglyceride-glucose related indices and all-cause and cause-specific mortality in the general population: a cohort study. Cardiovasc Diabetol. (2024) 23:286. doi: 10.1186/s12933-024-02390-0, PMID: 39113049 PMC11304911

[29] Cai XL Xiang YF Chen XF Lin XQ Lin BT Zhou GY . Prognostic value of triglyceride glucose index in population at high cardiovascular disease risk. Cardiovasc Diabetol. (2023) 22:198. doi: 10.1186/s12933-023-01924-2, PMID: 37537553 PMC10398968

[30] Xu X Huang R Lin Y Guo Y Xiong Z Zhong X . High triglyceride-glucose index in young adulthood is associated with incident cardiovascular disease and mortality in later life: insight from the CARDIA study. Cardiovasc Diabetol. (2022) 21:155. doi: 10.1186/s12933-022-01593-7, PMID: 35962377 PMC9375240

[31] Liang D Liu C Wang Y . The association between triglyceride-glucose index and the likelihood of cardiovascular disease in the U.S. population of older adults aged ≥ 60 years: a population-based study. Cardiovasc Diabetol. (2024) 23:151. doi: 10.1186/s12933-024-02248-5, PMID: 38702717 PMC11067197

[32] Sun M Guo H Wang Y Ma D . Association of triglyceride glucose index with all-cause and cause-specific mortality among middle age and elderly US population. BMC Geriatr. (2022) 22:461. doi: 10.1186/s12877-022-03155-8, PMID: 35643423 PMC9145102

[33] Chen Y Xie K Han Y Ju H Sun J Zhao X . The association between triglyceride-glucose index and its combination with systemic inflammation indicators and all-cause and cardiovascular mortality in the general US population: NHANES 1999-2018. Lipids Health Dis. (2024) 23:289 doi: 10.1186/s12944-024-02277-9, PMID: 39256829 PMC11386374

[34] Wei X Min Y Song G Ye X Liu L . Association between triglyceride-glucose related indices with the all-cause and cause-specific mortality among the population with metabolic syndrome. Cardiovasc Diabetol. (2024) 23:134. doi: 10.1186/s12933-024-02215-0, PMID: 38658993 PMC11044377

[35] Du L Xu X Wu Y Yao H . Association between the triglyceride glucose index and cardiovascular mortality in obese population. NutrMetab Cardiovasc Dis. (2024) 34:107–11. doi: 10.1016/j.numecd.2023.08.007, PMID: 37949711

[36] Gao B Yang C Wu G Zhao G Huang J Wang L . The triglyceride glucose index was nonlinearly associated with all-cause mortality in diabetic patients. NutrMetab Cardiovasc Dis. (2024) 34:2012–5. doi: 10.1016/j.numecd.2024.04.009, PMID: 38866610

